# Small Bowel Obstruction Caused by Obturator Hernia

**DOI:** 10.5334/jbsr.3715

**Published:** 2024-11-05

**Authors:** Olivier Colsoul, Adelard De Backer, Frederik Vandenbroucke

**Affiliations:** 1 Vrije Universiteit Brussel (VUB), Universitair Ziekenhuis Brussel (UZ Brussel), Department of Radiology, Laarbeeklaan 101, 1090 Brussels, Belgium

**Keywords:** CT, intestinal obstruction, obturator hernia

## Abstract

*Teaching point:* In an elderly patient with a history of atypical, intermittent medial thigh pain presenting with signs of intestinal obstruction, entrapment of bowel loops in an obturator hernia should be included in the differential diagnosis and should be further elaborated by computed tomography (CT).

## Case History

**Figure 1:**
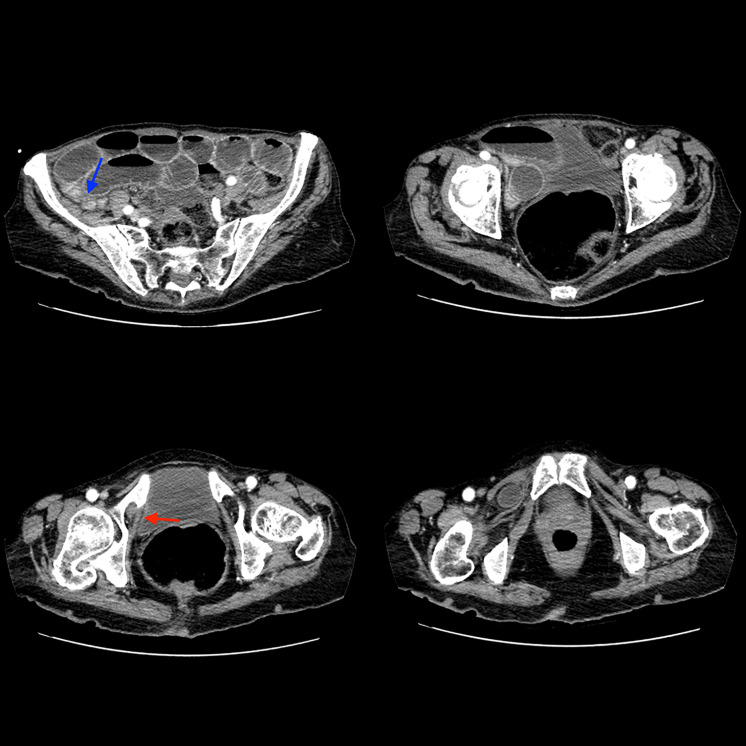
Axial images demonstrating entrapped obturator hernia and small bowel obstruction.

**Figure 2:**
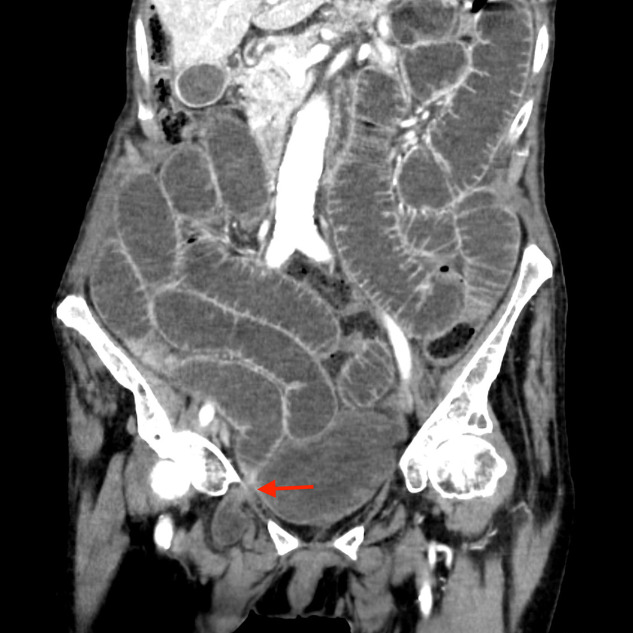
Coronal image demonstrating entrapped obturator hernia and small bowel obstruction.

**Figure 3:**
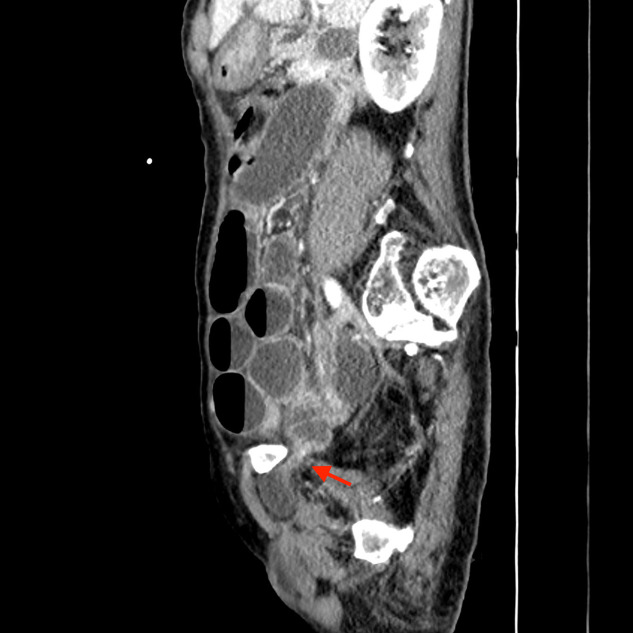
Sagittal image demonstrating entrapped obturator hernia and small bowel obstruction.

An 83‑year‑old man presented with acute onset of abdominal pain and vomiting. Medical history revealed intermittent episodes of medial thigh pain. Clinical examination showed abdominal distension without rebound tenderness. The most intense pain was located in the right lower abdomen. There was no palpable mass in the inguinal region. Contrast‑enhanced computed tomography (CT) showed dilated small bowel loops with fluid accumulation. A herniation of a small bowel loop through the right obturator foramen (Figure 1–3: red arrow) was observed extending inferiorly between the obturator externus and the pectineus muscle. Distal small bowel loops were collapsed (Figure 1: blue arrow). Signs of bowel ischemia and pneumoperitoneum were absent. Diagnosis of an entrapped obturator hernia resulting in small bowel obstruction was made. Lower midline infra‑umbilical laparotomy confirmed the obstruction caused by the obturator hernia. The entrapped distal ileal bowel segment was released. No resection of the entrapped small bowel loop was necessary.

## Comments

Obturator hernias (OH) are rare, constituting approximately 1% of all intra‑abdominal hernias. OH occur when the hernia sac descends through the obturator foramen where the obturator nerve and muscle pass [1].

Predisposing factors associated with OH include a wider pelvis, multiparity, and elderly women usually in conjunction with emaciation, cachexia, and chronic medical conditions resulting in increased intra‑abdominal pressure [1].

Due to its deep location, palpation of the sac of an obturator hernia in asymptomatic patients is extremely difficult and, therefore, the diagnosis is often initially clinically overlooked. In some patients, a palpable mass in the groin with the hip flexed and laterally rotated may be noted. Abdominal pain is the most frequent symptom, but is nonspecific, and most patients present with mechanical bowel obstruction at the time of diagnosis due to incarceration. The pathognomonic Howship–Romberg sign, described as pain exacerbated by extension, abduction, and internal rotation of the hip due to compression of the obturator nerve, is present in approximately half of the patients; however, its absence cannot reliably exclude OH. In other patients, loss of the adductor reflex (Hannington–Kiff sign) may be present. Some patients may experience atypical medial thigh pain and mild abdominal distention [1].

CT is the key imaging modality to detect OH. OH typically contain a loop of small bowel protruding through the obturator canal, with a hernia sac descending between the pectineus and obturator muscle, often displacing these muscles. If complicated by incarceration, small bowel obstruction and signs of bowel ischemia and perforation may be present.

Surgical intervention is the definitive management of OH causing small bowel obstruction. The procedure involves the repair of the OH, inspection of incarcerated bowel for viability, and, if indicated, bowel resection [1].
